# ﻿Two new species of *Navicula* (Bacillariophyta) from Southeast Asia

**DOI:** 10.3897/phytokeys.190.78164

**Published:** 2022-02-18

**Authors:** Maxim S. Kulikovskiy, Dmitry A. Chudaev, Anton M. Glushchenko, Irina V. Kuznetsova, John Patrick Kociolek

**Affiliations:** 1 K.A. Timiryazev Institute of Plant Physiology RAS, IPP RAS, 35 Botanicheskaya St., Moscow, 127276, Russia K.A. Timiryazev Institute of Plant Physiology RAS, IPP RAS Moscow Russia; 2 Department of Mycology and Algology, Faculty of Biology, M.V. Lomonosov Moscow State University, Leninskie Gory 1, building 12, Moscow, 119234, Russia M.V. Lomonosov Moscow State University Moscow Russia; 3 Museum of Natural History, Boulder, Colorado, 80309, USA Museum of Natural History Boulder United States of America; 4 Department of Ecology and Evolutionary Biology, University of Colorado, Boulder, Colorado, 80309, USA University of Colorado Boulder United States of America

**Keywords:** Diatoms, distribution, freshwaters, morphology, *Navicula*, new species, Southeast Asia, taxonomy

## Abstract

We present light and scanning electron microscopical observations on two new species of *Navicula* Bory sensu stricto from Southeast Asia. *Naviculawinoniformis* Chudaev, Glushchenko, Kulikovskiy & Kociolek, **sp. nov.** differs from taxa with similar outline and size by the combination of simple drop-like external raphe endings deflected to the primary valve side, presence of well-developed external longitudinal grooves and relatively low lineolae density. *Naviculasparsilineolata* Chudaev, Glushchenko, Kulikovskiy & Kociolek, **sp. nov.** can be discriminated from the taxa of similar valve shape and size by consistently lower lineolae density, and from the majority of them also by the structure of external proximal raphe endings with small projections in proximal parts and larger triangular insertions in distal parts. Some remarks on *Navicula* species diversity and its distribution in the Southeast Asia are given.

## ﻿Introduction

This investigation continues our description of new species from the genus *Navicula* Bory from Southeast Asia. With both freshwater and marine representatives, the genus *Navicula* Bory is the most taxon-rich genus of all diatoms (Kulikovskiy et al. 2016). However, the number of species included in this genus has actually been decreasing over the last 20 years mainly because of the description of new genera that include species formerly in in *Navicula*. The genus *Navicula* is easily distinguished from many other diatom genera by its lineolate uniseriate striae (Kulikovskiy et al. 2016). In some areas some morphologically unusual groups of species from the genus *Navicula* have been recognized, such as species with hyaline area on valve face. An example of this is *Naviculalacusbaicali* Skvortzov & Meyer from ancient Lake Baikal (Kulikovskiy et al. 2012). This group of *Navicula* formed species-flock (e.g. Kociolek et al. 2017); this is a group of closely-related taxa originated from single parent by explosive radiation (see Kociolek et al. 2019). New species from the genus *Navicula* are described often, indicating we are still gaining a better understanding of the real diversity in this genus. The taxonomy of some species in the genus is in need of clarification as well (Witkowski et al. 2010; Kulikovskiy et al. 2016).

Southeast Asia is a very interesting region for aquatic protist diversity, yet studies from the region are relatively few. More recently, we have been investigating diatoms and many other organisms of Southeast Asia (Glushchenko et al. 2021), including new genera and species, for example from Laos and Vietnam (e.g. Kulikovskiy et al. 2015, 2018, 2019; Glushchenko et al. 2016, 2017, 2018, 2019, 2020; Liu et al. 2018; Kezlya et al. 2020). A comprehensive review of diatom studies was summarized in Glushchenko et al. (2021). A few publications were dedicated to the diversity of new species from the genus *Navicula* (Chudaev et al. 2018; Kulikovskiy et al. 2020a,b, 2021) of Southeast Asia. Continuation of this work is important for understanding the diversity of the genus *Navicula* and diatoms as a whole from such an interesting region. Freshwater ecosystems from this region will need investigation in future for water quality assessment, using modern methods in ecological monitoring like DNA barcoding (Rimet et al. 2019). But a primary understanding of the biodiversity of the region is a necessary first step for future investigations and applying new methods.

The aim of this publication is to present a morphological investigation using light and scanning electron microscopy to describe two new diatom species of the genus *Navicula* from Southeast Asia.

## ﻿Materials and methods

Samples from Vietnam were collected by M.S. Kulikovskiy and E.S. Gusev during expeditions organized and permitted by the Joint Russian-Vietnam Tropical Centre, Ecolan 1.2 and 3.2 projects. The sample from Laos was collected by E.L. Konstantinov during an expedition of Kaluga and Laos Joint Universities (Russia and Laos).

A list of all samples examined in this study with their geographic positions is presented in Table 1. Water mineralization and temperature measurements were performed using the Hanna Combo (HI 98129) device, Hanna Instruments, Inc., USA. Material was collected with a pipette into 15- and 50-ml polymer test tubes. Samples were fixed with 37% formaldehyde.

**Table 1.** List of samples examined in this study. Geographic locality of samples and measured parameters indicated.

Slide	Locality	Habitat	Coordinates	Altitude, m	t, °C	pH	Conductivity, µS cm^-1^	Coll. date
**Vietnam**								
00269	Lâm Đồng Province, Da Tien Reservoir	benthos	11°58.816'N, 108°26.987'E	1503	21.5	6.4	81	21.06.2012
00318	Khánh Hòa Province, Suối Dầu River	benthos	12°06.768'N, 108°59.888'E	275	24	6.7	92	02.07.2012
00323		periphyton						
00326	Khánh Hòa Province, Suối Tiên River	benthos	12°12.199'N, 109°01.694'E	68	26	6.9	101	02.07.2012
00328		periphyton						
02079	Lào Cai Province, neat the Sa Pa Town, Mường Hoa River	periphyton	22°15.415'N, 103°8.883'E	887	25.5	8.4	204	10.05.2015
03572	Khánh Hòa Province, Suối Dầu Reservoir	benthos	12°09.900'N, 109°03.200'E	36	31.4	7.3	84	10.08.2010
03773	Khánh Hòa Province, Suối Dầu River	periphyton	12°06.768'N, 108°59.891'E	275	25	6.7	119	28.05.2012
04853	Khánh Hòa Province, Cái River	plankton	12°15.983'N, 109°06.517'E	13	31.6	7.0	40	17.04.2010
**Laos**								
00956	Vientiane Province, Van Vieng District, Nam Lik Village, Nam Lik River	benthos	18°36.808'N, 102°24.605'E	196	23.5	6.9	98	24.11.2011
00962	Champasak Province, Bolaven Plateau, near the Pakse Town, unnamed waterfall	benthos	15°16.616'N 106°19.935'E	1149	24.5	6.9	84	30.11.2011
01621	Champasak Province, Bolaven Plateau, near the Paksong Town, Tad Yueang Waterfall	benthos	13°57.266'N 105°54.890'E	78	26.2	7.1	96	01.12.2011

The samples were treated with 10% hydrochloric acid to remove carbonates and washed several times with deionized water for 12 h. Samples were subsequently boiled in concentrated hydrogen peroxide (≈ 37%) to dissolve organic matter. They were washed again with deionized water four times at 12 h intervals. After decanting and filling with deionized water up to 100 ml, the suspension was spread onto coverslips and left to dry at room temperature. Permanent diatom preparations were mounted in Naphrax (refraction index = 1.73). Light microscopic (LM) observations were performed with a Zeiss Axio Scope A1 microscope equipped with an oil immersion objective (× 100, n.a. 1.4, differential interference contrast [DIC]) and Axiocam ERc 5s camera (Zeiss). Valve ultrastructure was examined by means of scanning electron microscope JEOL JSM-6380LA (JEOL Ltd., Japan) operating at 20 kV and 8 mm working distance (Faculty of Biology, M.V. Lomonosov MSU, Moscow). For scanning electron microscopy (SEM), parts of the suspensions were fixed on aluminium stubs after air-drying. The stubs were sputter coated with 50 nm of gold in an Eiko IB 3.

Fixed material and slides are deposited in the collection of Maxim Kulikovskiy at the Herbarium of the Institute of Plant Physiology Russian Academy of Science, Moscow, Russia. Isotypes are deposited in Diatom collection of the Department of Mycology and Algology, Faculty of Biology, M.V. Lomonosov Moscow State University, Moscow, Russia.

## ﻿Results

### ﻿ Navicula winoniformis

Taxon classificationPlantaeNaviculalesNaviculaceae

Chudaev, Glushchenko, Kulikovskiy & Kociolek sp. nov.

Figs 1 , 2

Navicula gottlandica sensu Lee, 2012 (fig. 4E) (Algal Flora of Korea vol. 3, num. 8).

#### Holotype.

**Figure 1.**
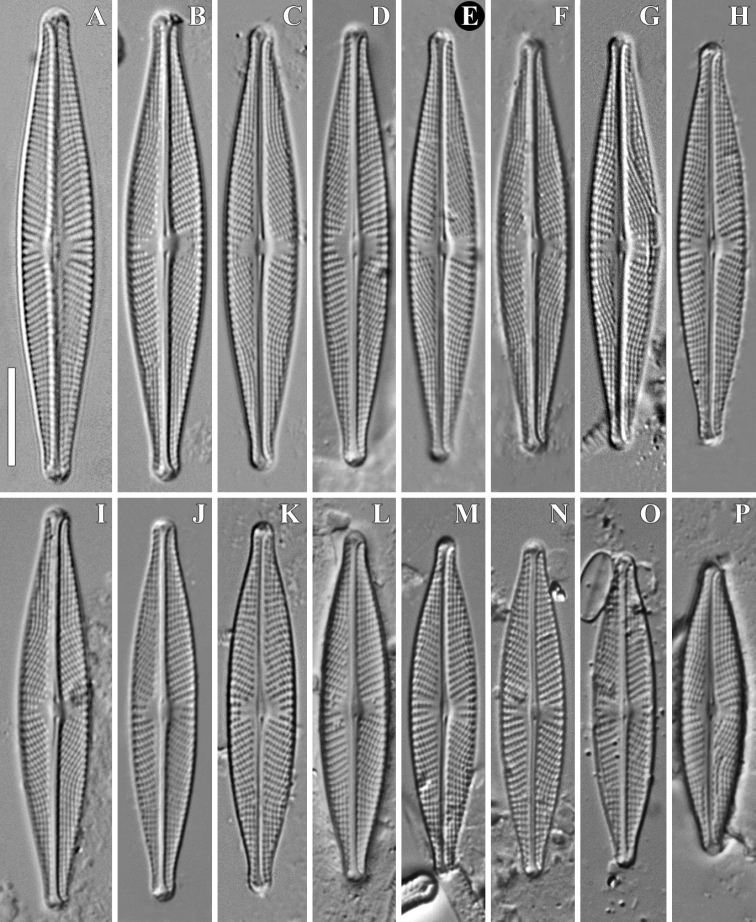
**A–P***Naviculawinoniformis* Chudaev, Glushchenko, Kulikovskiy & Kociolek, sp. nov. LM, DIC. Size diminution series. Vietnam, slides no 00269 (**A**), 00318 (**L, P, N**), 00323 (**I**), 00326 (**C**), 00328 (**B**), 02079 (**O**), 03572 (**E, F, H, K, M**), 03773 (**D**), 04853 (**J**). Laos, slide no 00956 (**G**). Holotype (**E**). Scale bar: 10 μm.

Slide 03572 in collection of Maxim Kulikovskiy at the Herbarium of the Institute of Plant Physiology Russian Academy of Science, Moscow, Russia, represented here by Fig. 1E.

#### Isotype.

Slide MW-D 898s1 in Diatom collection of the Department of Mycology and Algology, Faculty of Biology, M.V. Lomonosov Moscow State University, Moscow, Russia.

#### Type locality.

Vietnam. Khánh Hòa Province, Suối Dầu Reservoir, benthos, 12°09.900'N, 109°03.200'E, 36 m elev., leg. E.S. Gusev, 10.08.2010.

#### Description.

**LM** (Fig. 1A–P). Valves narrowly lanceolate with rostrate to subcapitate apices, length 29.7–49.0 μm, width 6.5–8.0 μm (n=32). Axial area narrow, slightly widening towards valve centre, central area transversely expanded, with irregular border due to unequal shortening of central striae, usually occupying about 1/2 of valve width. Striae radiate, becoming strongly convergent at the valve ends, 12–14/10 μm. Lineolae easy to resolve in light microscope, 24–27/10 μm. Raphe filiform to narrowly lateral, terminal fissures deflected to the secondary valve side, central pores straight or very slightly deflected to primary valve side, not close standing.

**Figure 2.**
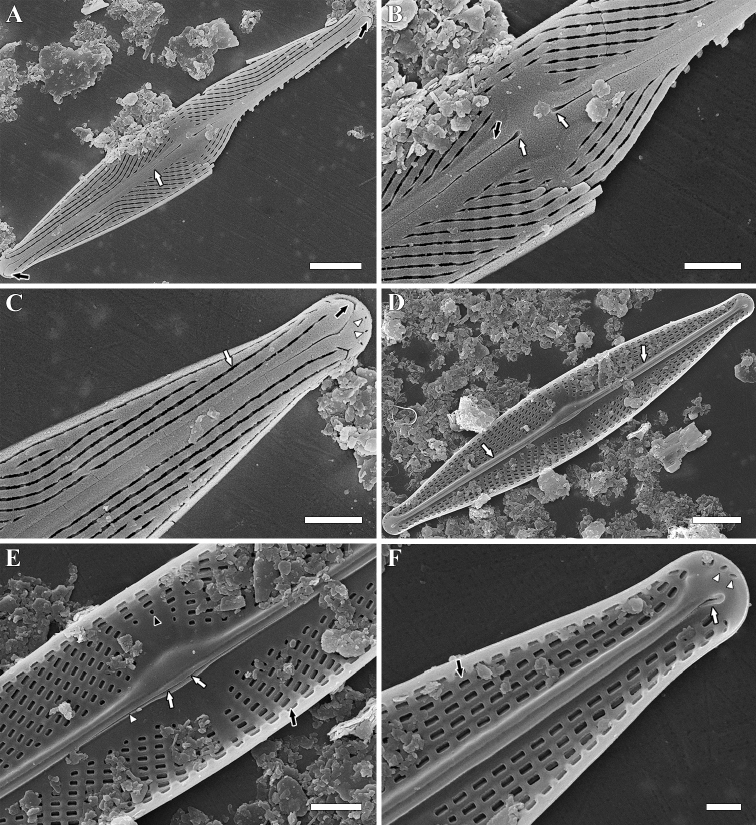
**A–F***Naviculawinoniformis* Chudaev, Glushchenko, Kulikovskiy & Kociolek, sp. nov. SEM. Sample no 03572. **A–C** external views **D–F** internal views **A, D** the whole valve **B, E** central area **C, F** valve end. **A** black arrows show the terminal raphe fissures. White arrow shows the indistinct kink of raphe branch **B** white arrows show central raphe pores. Black arrow shows the very slightly elevated raphe-sternum **C** black arrow shows the terminal raphe fissure. White arrow shows the longitudinal grooves where areola openings are located. White arrowheads show the small isolated apical areolae **D** white arrows show the accessory rib **E** black arrow shows the rectangular areola opening. Black arrowhead shows the circular areola opening. White arrows show the central raphe endings. White arrowhead shows the raphe slit **F** black arrows show the stria which are slightly wider than virgae. White arrow shows the helicroglossa. White arrowheads show the small isolated apical areolae. Scale bars: 5 μm (**A**); 2 μm (**B–E**); 1 μm (**F**).

**SEM, external view** (Fig. 2A–C). Areola openings apically elongate, lying in distinct longitudinal grooves (Fig. 2C, white arrow). Four small isolated areolae with almost circular openings present at valve apices on valve mantle (Fig. 2C, white arrowheads). Raphe-sternum elevated very slightly in valve centre (Fig. 2B, black arrow). Central pores drop-like without any projections, deflected slightly to primary valve side (Fig. 2B, white arrows). Terminal fissures hooked to secondary valve side (Fig. 2A, C, black arrows). Raphe branches with indistinct kink in proximal parts (Fig. 2A, white arrow).

**SEM, internal view** (Fig. 2D–F). Striae slightly wider than virgae, lie at same level as latter, no transapical grooves formed (Fig. 2E, F, black arrows). Internal areola openings rectangular (Fig. 2F, black arrow) or circular (near central area) (Fig. 2E, black arrowhead), wider than external ones. Hymenes were not preserved during the material treatment. Four to five small isolated areolae present at each apex (Fig. 2F, white arrowheads). Raphe slit opens obliquely to secondary side and visible in proximal (Fig. 2E, white arrowhead) and distal (Fig. 2F, white arrow) parts only. Raphe-sternum very narrow, widened in centre, separated from accessory rib by longitudinal groove. Accessory rib well-developed (Fig. 2D, white arrows), widened unilaterally in valve centre and at valve apices. Central raphe endings straight, simple, connected by thin indistinct furrow (Fig. 2E, white arrows). Distal raphe endings well-developed helictoglossae deflected to secondary valve side (Fig. 2F, white arrow).

#### Etymology.

Specific epithet is given due to similarity of new species to *Naviculawinona* Bahls.

#### Distribution.

Vietnam. Type locality (Suối Dầu Reservoir, slide no 03572). Suối Dầu River (slide no 00318, 00323, 03773), Suối Tiên River (slide no 00326, 00328), Cái River (slide no 04853), Mường Hoa River (slide no 02079), Da Tien Reservoir (slide 02069). Laos, Nam Lik River (slide no 00956).

#### Ecology.

*Naviculawinoniformis* sp. nov. was found in a the reservoirs, and in rivers and waterfalls with different conductivity and pH values (Table 1). Below are the dominant species found in the samples along with *N.winoniformis* sp. nov.:

Sample 03572: *Neidiumgracile* Hustedt, *Encyonopsisfonticola* (Hustedt) Krammer. Sample 00956: *Oricymbavoronkinae* Glushchenko, Kulikovskiy & Kociolek, *Pinnulariasikkimensis* S.K. Das, C. Radhakrishnan, Kociolek & Karthick, *Pinnulariastricta* Hustedt, *Luticolamuticoides* (Hustedt) D.G. Mann.

Sample 00318: *Hydroseratriquetra* Wallich, *Diadesmisconfervacea* Kützing, *Luticolanipkowii* (Meister) Glushchenko & Kulikovskiy.

Sample 00323: *Gomphonemadalatica* Glushchenko, Kulikovskiy & Kociolek, *Eunotiaindosinica* Glushchenko & Kulikovskiy.

Sample 03773: *Eunotiaindomalaica* Glushchenko, Kulikovskiy & Kociolek, *O.voronkinae*.

Sample 00326: *Gomphonemasubventricosum* Hustedt, *D.confervacea*, *Oricymbaperjaponica* (Krammer & Lange-Bertalot) Kulikovskiy, Glushchenko & Kociolek, *Rhopalodiagibba* (Ehrenberg) O. Müller, *Encyonemajavanicum* (Hustedt) D.G. Mann.

Sample 00328: *Placoneisparaundulata* T. Ohtsuka, *E.javanicum*, *Eunotialaoarcus* Glushchenko, Kulikovskiy & Kociolek.

Sample 04853: *D.confervacea*, *Platessaoblongella* (Østrup) Wetzel, Lange- Bertalot & Ector, *Luticolataylorii* Levkov, Metzeltin & Pavlov, *N.gracile*, *E.javanicum*, *O.voronkinae*, *Frustuliamagaliesmontana* Cholnoky.

Sample 02079: *P.oblongella*, *Reimeriasinuata* (Gregory) Kociolek & Stoermer, *E.javanicum*, *Encyonemaleei* Krammer.

Sample 00269: *G.dalatica*, *Adlafialamdongiensis* Glushchenko, Kulikovskiy & Kociolek, *Kobayasiellalamii* Glushchenko, Kulikovskiy & Kociolek.

### ﻿ Navicula sparsilineolata

Taxon classificationPlantaeNaviculalesNaviculaceae

Chudaev, Glushchenko, Kulikovskiy & Kociolek sp. nov.

Figs 3 , 4

#### Holotype.

**Figure 3.**
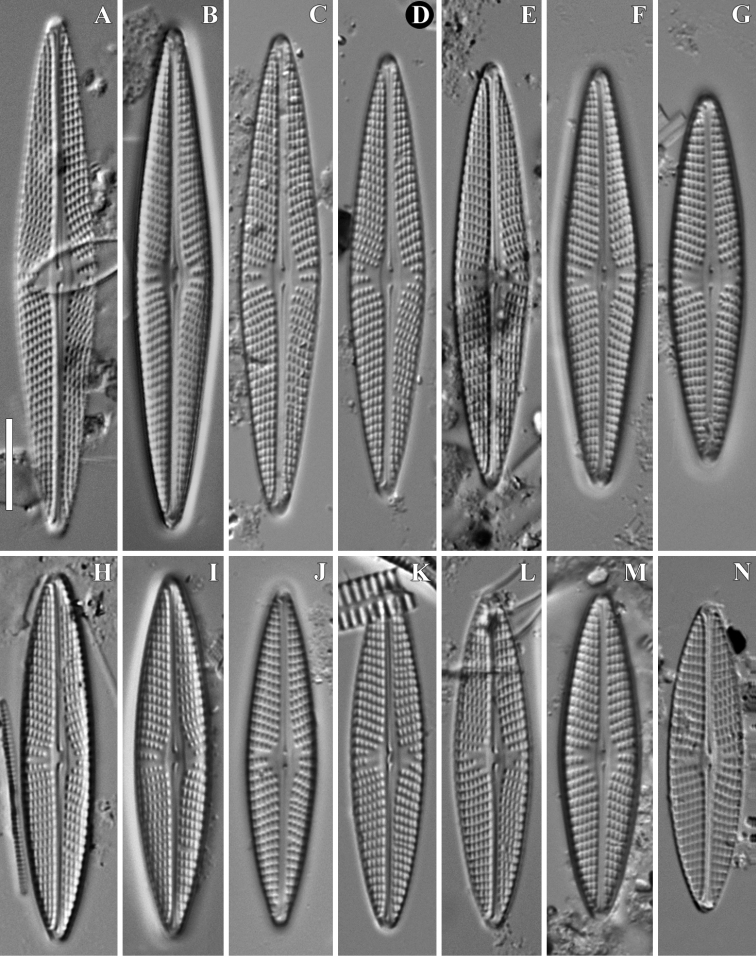
**A–N***Naviculasparsilineolata* Chudaev, Glushchenko, Kulikovskiy & Kociolek, sp. nov. LM, DIC. Size diminution series. Laos, slides no 00962 (**A, B, F, C–E, G, I, J, L, M, N**), 01621 (**H, K**). Holotype (**D**). Scale bar: 10 μm.

Slide 00962 in collection of Maxim Kulikovskiy at the Herbarium of the Institute of Plant Physiology Russian Academy of Science, Moscow, Russia, represented here by Fig. 3D.

#### Isotype.

Slide MW-D 899s1 in Diatom collection of the Department of Mycology and Algology, Faculty of Biology, M.V. Lomonosov Moscow State University, Moscow, Russia.

#### Type locality.

Laos. Champasak Province, Bolaven Plateau, near the Pakse Town, unnamed waterfall, benthos, 15°16.616´N, 106°19.935´E, 1149 m elev., leg. E.L. Konstantinov, 30.11.2011.

#### Description.

**LM** (Fig. 3A–N). Valves lanceolate with acutely rounded, non-protracted apices, length 33.9–56.5 μm, width 7.7–9.1 μm (n=31). Axial area narrow, widening towards valve centre, slightly wider on the secondary valve side, central area more or less round, occupying 1/3–1/2 of valve width, margins of raphe-sternum clearly visible as longitudinal lines at the central area. Striae radiate, sometimes (usually in larger valves) becoming parallel at the valve ends, 9.3–10.5/10 μm. Lineolae easy to resolve in light microscope, 17.5–19.7/10 μm. Raphe filiform to narrowly lateral, terminal fissures barely visible, deflected to the secondary valve side, central pores deflected to secondary valve side.

**Figure 4.**
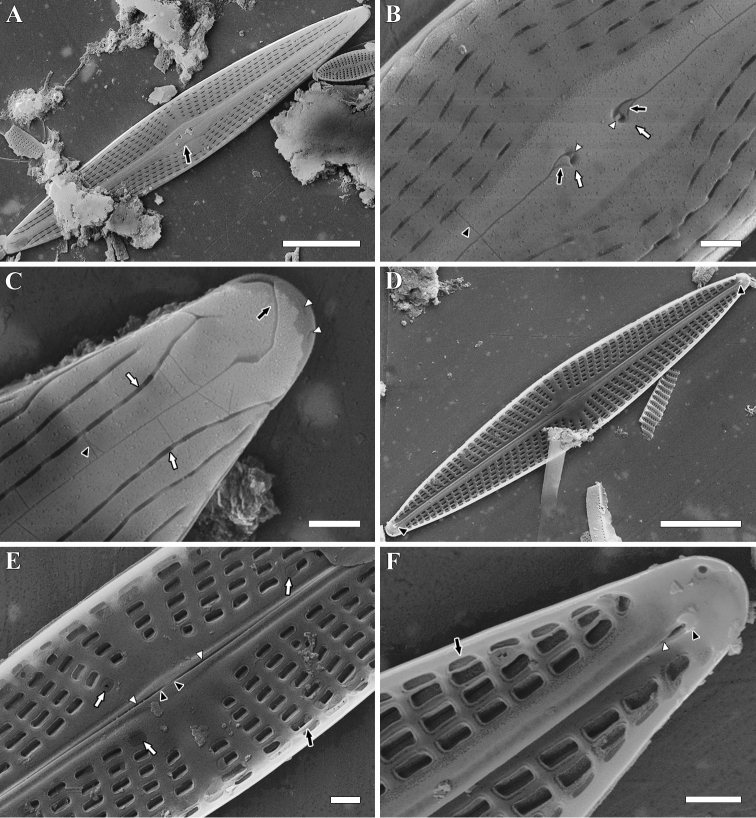
**A–F***Naviculasparsilineolata* Chudaev, Glushchenko, Kulikovskiy & Kociolek, sp. nov. SEM. Sample no 00962. **A–C** external views **D–F** internal views **A–C** external views **D–F** internal views **A, D** the whole valve **B, E** central area **C, F** valve end **A** black arrow shows the very slightly elevated raphe-sternum **B** white arrows show the drop-like central pores. Black arrows show the larger triangular insertion in distal part. White arrowheads show the small projections in proximal part. Black arrowhead shows the transapical sutures **C** black arrow shows the terminal raphe fissure. White arrows show the longitudinal grooves where areola openings are located. Black arrowhead shows the transapical sutures. White arrowheads show the small isolated apical areolae **D** black arrowheads show the helictoglossae **E** black arrow shows the stria which slightly wider than virgae. White arrows show the areola openings occluded with hymenes. Black arrowheads show the central raphe endings. White arrowheads show the raphe slits **F** black arrow show the stria which are wider than virgae. Black arrowhead shows the helictoglossa. White arrowhead shows the raphe slit. Scale bars: 10 μm (**A, D**); 1 μm (**B, C, E, F**).

**SEM, external view** (Fig. 4 A–C). Areolae apically elongate, areolae openings lie in shallow apical grooves more clearly expressed near valve apices (Fig. 4C, white arrows). At each apex two small isolated apical areolae present with shorter slits oriented obliquely or subparallel to valve margin (Fig. 4C, white arrowheads). Raphe-sternum elevated slightly above valve surface in centre, widened, with asymmetrical margins (Fig. 4A, black arrow). Its primary margin slightly convex, secondary margin slightly concave or straight. Central pores drop-like, deflected to secondary side. Each pore possesses small projection in proximal part (Fig. 4B, white arrowheads) and larger triangular insertion in distal part (Fig. 4B, black arrows). Terminal fissures hooked to secondary side (Fig. 4C, black arrow), proximal parts of fissures dilated. On axial area are present thin transapical sutures (Fig. 4B, C, black arrowheads).

**SEM, internal view** (Fig. 4D–F). Striae wider than virgae, lie at same level as latter, no transapical grooves formed (Fig. 4E, F, black arrows). Internal areola openings occluded with hymens (mostly destroyed) rectangular, wider than external ones (Fig. 4E, black arrows). Raphe slit opens obliquely to secondary side and visible in proximal and distal parts only (Fig. 4E, F, white arrowheads). Raphe-sternum very narrow, widened in centre, flanked with apical grooves from both sides. Accessory rib almost absent, especially in proximal part of valve. Central raphe endings straight, simple (Fig. 4E, black arrowheads). Distal raphe endings well-developed straight helictoglossae (Fig. 4D, F, black arrowheads).

#### Etymology.

The specific epithet refers to the comparatively low density of areolae in the new species.

#### Distribution.

Laos. Type locality (unnamed waterfall, slide no 00962) and Tad Yueang Waterfall (slide no 01621).

#### Ecology.

*Naviculasparsilineolata* sp. nov. was found in a the waterfalls with low conductivity and circumneutral pH values (Table 1). Below are the dominant species found in the samples along with *N.sparsilineolata* sp. nov:

Sample 00962: *Gomphonemacapitatum* Ehrenberg, *D.confervacea*, *Luticolaburmensis* Metzeltin & Levkov, *L.nipkowii*, *P.sikkimensis*.

Sample 01621: *L.burmensis*, *L.nipkowii*, *P.sikkimensis*, *Platessaoblongella* (Østrup) Wetzel, Lange- Bertalot & Ector.

## ﻿Discussion

The new species *Naviculawinoniformis* sp. nov. is similar to a few previously described species. These include *N.cryptocephala* Kützing, 1844, *N.krammerae* Lange-Bertalot, 1996, *N.densilineolata* (Lange-Bertalot) Lange-Bertalot, 1993, *N.wildii* Lange-Bertalot, 1993, *N.insulsa* Metzeltin & Lange-Bertalot, 1998, *N.oetzvallensis* Lange-Bertalot in Werum & Lange-Bertalot, 2004, *N.winona* Bahls, 2012 and *N.praeterita* Hustedt, 1945. A summary comparison of these taxa is presented in Table 2.

**Table 2.** Morphometric features of *Naviculawinoniformis* Chudaev, Glushchenko, Kulikovskiy & Kociolek sp. nov. and comparison with similar taxa.

Taxon	Outline	Valve ends	Valve length, μm	Valve width, *μm*	Striae in 10 μm	Areolae in 10 μm	References
*N.winoniformis* sp. nov.	narrowly lanceolate	rostrate to subcapitate	29.7–49.0	6.5–8.0	12–14	24–27	This study
*N.cryptocephala*	lanceolate to narrowly lanceolate	gradually narrowing or weakly rostrate, subcapitate to obtusely rounded	20–40	5–7	14–18	40–44	Lange-Bertalot 2001; Jüttner et al. 2020
*N.krammerae*	lanceolate	protracted and beak-like, neither distinctly nor acutely rounded	28–36	6.0–7.5	13–14	28–31	Lange-Bertalot 2001
*N.densilineolata*	narrowly lanceolate	almost acutely rounded	28–60	6.0–7.5	10–13	27–30	Lange-Bertalot 2001
*N.wildii*	(narrow) lanceolate	gradually narrowing to obtusely rounded, very rarely almost imperceptibly protracted	23–50	5.5–7.5	11.0–12.5	ca. 35	Lange-Bertalot 2001; Kulikovskiy et al. 2016
*N.insulsa*	rather linear-lanceolate	protracted, subcapitate to obtusely rounded	40–54	6–7	14–16	25–27	Metzeltin and Lange-Bertalot 1998
*N.oetzvallensis*	narrowly lanceolate to linear-lanceolate	weakly protracted	30–44	7.0–7.5	10.5–12.5	28–30	Werum and Lange-Bertalot 2004
*N.winona*	narrowly lanceolate	gradually attenuated and very narrow subcapitate	39–52	7.2–8.2	12–13	24–28	Bahls 2012
*N.praeterita*	lanceolate	rostrate-subcapitate	25–40	5.5–8.5	12–14	22–25	Lange-Bertalot 2001

*N.cryptocephala* differs from *N.winoniformis* sp. nov. by having lineolae difficult to resolve in LM (40–44/10 μm) and denser striae (14–18/10 μm), no external longitudinal grooves are formed in *N.cryptocephala* and the accessory rib is not unilaterally widened internally (Lange-Bertalot 2001; Jüttner et al. 2020). *N.krammerae* has higher lineolae density (28–31/10 μm) and straight central pores (Lange-Bertalot 2001). *N.densilineolata* possesses non protracted valve ends, no external longitudinal grooves, smaller central area, striae not distinctly convergent at the valve ends, central pores that are bent to the secondary side and denser lineolae (27–30/10 μm) (Lange-Bertalot 2001). *N.wildii* has a smaller central area, coarser striae (11.0–12.5/10 μm), lineolae difficult to resolve in LM, central pores that are bent to the secondary side, and no external longitudinal grooves are formed (Lange-Bertalot 2001). *N.insulsa* has denser striae (14–16/10 μm) and a rather linear-lanceolate valve outline (Metzeltin, Lange-Bertalot 1998). *N.oetzvallensis* has coarser striae (10.5–12.5/10 μm), denser areolae (28–30/10 μm), no external longitudinal grooves and its central pores are broadly expanded and hooked to secondary side (Werum and Lange-Bertalot 2004). *N.winona* differs by gradually attenuated valve ends and proximal raphe ends deflected to the secondary valve side (Bahls 2012). Though *N.praeterita* is quite similar to *N.winoniformis* in LM appearance (Lange-Bertalot 2001), it differs clearly under SEM by the structure of external proximal raphe endings, possessing small projections and larger insertions, and by the absence of distinct external longitudinal grooves.

At the moment *Naviculawinoniformis* sp. nov. is abundant and common in rivers and waterbodies of Khánh Hòa Province in Vietnam. Additionally, this species was found in Lâm Đồng Province, neighboring the previous one. We also found this species in a northern province in Vietnam – Lào Cai. Possibly this species is distributed in China too. We found this species only in the River Nam Lik in Laos. We believe that this species is widespread in freshwater systems of Vietnam, Laos and, possibly, China. Additionally, the species was found in Korea as *Naviculagottlandica* Grunow (Lee 2012).

*Naviculasparsilineolata* sp. nov. described herein, shows some similarity with species such as *Naviculapseudolanceolata* Lange-Bertalot, 1980, *Naviculaoppugnata* Hustedt, 1945, *Naviculajohncarteri* D.M. Williams, 2001 (syn. *N.concentrica* Carter in Carter & Bailey-Watts, 1981), *Naviculatrophicatrix* Lange-Bertalot in Lange-Bertalot & Metzeltin, 1996, *Naviculasancti-naumii* Levkov & Metzeltin, 2007, *Naviculaweberi* Bahls, 2012 (Table 3).

**Table 3.** Morphometric features of *Naviculasparsilineolata* Chudaev, Glushchenko, Kulikovskiy & Kociolek sp. nov. and comparison with similar taxa.

Taxon	Outline	Valve ends	Valve length, μm	Valve width, *μm*	Striae in 10 μm	Areolae in 10 μm	References
*N.sparsilineolata* sp. nov.	lanceolate	acutely rounded, non protracted	33.9–56.5	7.7–9.1	9.3–10.5	17.5–19.7	This study
*N.pseudolanceolata*	lanceolate to rhombic-lanceolate	gradually narrowed	28.7–50.0	7.0–9.8	9.5–11.2	22.0–25.7	Lange-Bertalot 2001; Chudaev and Gololobova 2016
*N.oppugnata*	lanceolate to linear-lanceolate	usually obtusely rounded	30–60	8.5–12.0	7–12	ca. 24	Lange-Bertalot 2001
*N.johncarteri*	lanceolate	gradually narrowing to a wedge, neither distinctly acutely nor obtusely rounded	40–75	9–12	8–10	ca. 25	Lange-Bertalot 2001
*N.trophicatrix*	lanceolate to rhombic-lanceolate	gradually narrowing to a wedge, neither distinctly acutely nor obtusely rounded	25–50	7.5–10.0	11–13	21–24	Lange-Bertalot 2001
*N.sancti-naumii*	strictly lanceolate	more or less acutely rounded	28–48	7.0–8.5	10–11	28–30	Levkov et al. 2007
*N.weberi*	elliptic-lanceolate to broadly lanceolate	obtusely rounded	29–57	7.3–10.3	9–10	ca. 24	Bahls 2012

*Naviculapseudolanceolata* differs from *N.sparislineolata* by having denser lineolae (22.0–25.7/10 μm, Lange-Bertalot 2001; Chudaev and Gololobova 2016) and simple drop-like central pores without any projections (Chudaev and Gololobova 2016, pl. 202, fig. 19). *Naviculaoppugnata* is characterized by denser lineolae (c. 24/10 μm), and more obtusely rounded valve ends, raphe-sternum appears not elevated externally in light micrographs (Lange-Bertalot 2001) and these features differentiate it from our new species. *Naviculajohncarteri* differs from *N.sparislineolata* by possessing wider valves (9–12 μm) with finer lineolae (c. 25/10 μm) and central pores without larger triangular insertions (as *N.concentrica* J. Carter in Carter & Bailey-Watts; see Lange-Bertalot 2001, pl. 72, fig. 6). Though *Naviculatrophicatrix* is similar to *N.sparsilineolata* in external central pores structure, it has less curved terminal fissures (Lange-Bertalot 2001, pl. 66, fig. 1), denser striae (11–13/10 μm) and lineolae (21–24/10 μm) and rather rhombic-lanceolate than lanceolate valve outline (Lange-Bertalot 2001). In *Naviculasancti-naumii* central pores have only small proximal projections and no external longitudinal grooves are formed (Levkov et al. 2007, pl. 47, figs 3–5); areola density in this species is higher (28–30/10 μm), all features that differentiate this species from *N.sparislineolata*. *Naviculaweberi* also have denser lineolae (c. 24/10 μm) and striae are distinctly convergent at valve ends (Bahls 2012), two features that distinguish this diatom from *N.sparislineolata*. *Naviculasparsilineolata* sp. nov. was found in two waterfalls in Laos. These waterfalls are 150 km from each other and situated in one Province, namely Champasak. It is possible that this species has more widespread distribution in Southeast Asia.

Description of these two new species shows that the diatom flora of Southeast Asia is a site of biodiversity discovery. The two species described herein are different from some other taxa previously described by us in that they are smaller and lack such prominent morphological features (see Kulikovskiy et al. 2020a,b). *Naviculababeiensis* Chudaev, Glushchenko, Kulikovskiy & Kociolek and *Naviculapseudokuseliana* Chudaev, Glushchenko, Kulikovskiy & Kociolek are smaller and have no special morphological peculiarities like the two species described here (Kulikovskiy et al. 2021). Two previously described species, *Naviculagogorevii* Chudaev, Glushchenko, Kulikovskiy & Kociolek and *Naviculadavidovichii* Chudaev, Glushchenko, Kulikovskiy & Kociolek, are characterized by having large valves and large areolae in the striae. *Naviculadavidochii* is very interesting and characterized by having a valve shape that is more typical for the genus *Pinnularia* Ehrenberg. Even within the genus *Naviculasensu stricto* we see a diversity of ultrastructural features. And representatives with these different morphologies are present in Southeast Asia. More work is needed to sort out the morphological diversity within *Navicula*, to understand the phylogenetic relationships of these morphological groups, and to establish whether there are biogeographic patterns that correspond to the relationships.

In the book “The diatoms of Southeast Asia” we included widespread taxa from Southeast Asia (Glushchenko et al. 2021). *Navicula* species were detected from three countries including Vietnam, Laos and Cambodia. Widespread taxa are *Naviculaescambia* (Patrick) Metzeltin & Lange-Bertalot, 2007, *N.simulata* Manguin 1942, *N.nielsfogedii* Taylor & Cocquyt in Taylor, Cocquyt & Mayama, 2016, *N.heimansioides* Lange-Bertalot, 1993, *N.globuliferiformis* Lange-Bertalot 1993, *N.gondwana* Lange-Bertalot, 1993, *N.tripunctata* (O.F. Müller) Bory, 1822, *N.rostellata* Kützing, 1844, *N.subrhynchocephala* Hustedt, 1935, *N.ingapirca* U. Rumrich & Lange-Bertalot in U. Rumrich, Lange-Bertalot & M. Rumrich, 2000, *N.recens* (Lange-Bertalot) Lange-Bertalot in Krammer & Lange-Bertalot, 1985, *N.radiosa* Kützing, 1844, *N.avenacea* (Rabenhorst) Brébisson ex Grunow in Schneider, 1878, *N.angusta* Grunow, 1860, *N.namibica* Lange-Bertalot & U. Rumrich in Lange-Bertalot, 1993, *N.caterva* Hohn & Hellerman, 1963, *N.vandamii* Schoeman & Archibald, 1987, *N.germainii* Wallace, 1960, *N.amphiceropsis* Lange-Bertalot & U. Rumrich in U. Rumrich, Lange-Bertalot & M. Rumrich, 2000, *N.quasidisjuncta* Lange-Bertalot & U. Rumrich in U. Rumrich, Lange-Bertalot & M. Rumrich, 2000, *N.electrolytifuga* Lange-Bertalot & U. Rumrich in U. Rumrich, Lange-Bertalot & M. Rumrich, 2000 (Glushchenko et al. 2021).

As evident from the above list of known taxa, Southeast Asia includes many species described from other areas of the Southern Hemisphere, mainly from South America (Rumrich et al. 2000). *N.nielsfogedii* is a widespread taxon on the basis of its morphospecies taxonomy (Chudaev et al. 2020). Possibly, it is cryptic species that can be evident on the basis of molecular investigation in the future. However, if we use molecular methods we can find cryptic speciation. This occurs when some small morphological features (mainly in valve dimensions and shape) are evident in different populations (Chudaev et al. 2020; Glushchenko et al. 2021). Cryptic speciation is a phenomenon that is known in diatoms (Mann 1999); however we do not know precisely how extensively it occurs in different groups of diatoms (Maltsev et al. 2021). Cryptic speciation was shown in the genus *Navicula* by Poulíčková et al. (2010) on the basis investigation of *Naviculacryptocephala*. The same situation can be detected for *N.electrolytifuga* with populations from Laos, Cambodia and Vietnam (see Glushchenko et al. 2021: Pl. 86). Species such as *N.heimansioides*, *N.tripunctata*, *N.rostellata*, *N.subrhynchocephala*, *N.radiosa* are widespread across the Northern and Southern Hemispheres and found in different types of fresh waterbodies (Lange-Bertalot 2001; Kulikovskiy et al. 2016). Molecular investigation of these taxa will be important for the future research of their relationships and use in water quality analysis.

## Supplementary Material

XML Treatment for Navicula winoniformis

XML Treatment for Navicula sparsilineolata
